# Novel dual-pathogen multi-epitope mRNA vaccine development for Brucella melitensis and Mycobacterium tuberculosis in silico approach

**DOI:** 10.1371/journal.pone.0309560

**Published:** 2024-10-28

**Authors:** Yuejie Zhu, Juan Shi, Quan Wang, Yun Zhu, Min Li, Tingting Tian, Huidong Shi, Kaiyu Shang, Zhengwei Yin, Fengbo Zhang

**Affiliations:** 1 Department of Reproductive Assistance, Center for Reproductive Medicine, The First Affiliated Hospital of Xinjiang Medical University, Urumqi, Xinjiang, China; 2 The First Affiliated Hospital of Xinjiang Medical University, Urumqi, Xinjiang, China; 3 The Eighth Affiliated Hospital of Xinjiang Medical University, Urumqi, Xinjiang, China; 4 Xinjiang Uygur Autonomous Region Disease Prevention Control Center, Urumqi, Xinjiang, China; 5 Department of Clinical Laboratory, The First Affiliated Hospital of Xinjiang Medical University, Urumqi, Xinjiang, China; 6 State Key Laboratory of Pathogenesis, Prevention, Treatment of Central Asian High Incidence Diseases, The First Affiliated Hospital of Xinjiang Medical University, Urumqi, Xinjiang, China; 8th Medical Center of Chinese PLA General Hospital, CHINA

## Abstract

Brucellosis and Tuberculosis, both of which are contagious diseases, have presented significant challenges to global public health security in recent years. Delayed treatment can exacerbate the conditions, jeopardizing patient lives. Currently, no vaccine has been approved to prevent these two diseases simultaneously. In contrast to traditional vaccines, mRNA vaccines offer advantages such as high efficacy, rapid development, and low cost, and their applications are gradually expanding. This study aims to develop multi-epitope mRNA vaccines argeting Brucella melitensis and Mycobacterium tuberculosis H37Rv (L4 strain) utilizing immunoinformatics approaches. The proteins Omp25, Omp31, MPT70, and MPT83 from the specified bacteria were selected to identify the predominant T- and B-cell epitopes for immunological analysis. Following a comprehensive evaluation, a vaccine was developed using helper T lymphocyte epitopes, cytotoxic T lymphocyte epitopes, linear B-cell epitopes, and conformational B-cell epitopes. It has been demonstrated that multi-epitope mRNA vaccines exhibit increased antigenicity, non-allergenicity, solubility, and high stability. The findings from molecular docking and molecular dynamics simulation revealed a robust and enduring binding affinity between multi-epitope peptides mRNA vaccines and TLR4. Ultimately, Subsequently, following the optimization of the nucleotide sequence, the codon adaptation index was calculated to be 1.0, along with an average GC content of 54.01%. This indicates that the multi-epitope mRNA vaccines exhibit potential for efficient expression within the *Escherichia coli(E*. *coli)* host. Analysis through immune modeling indicates that following administration of the vaccine, there may be variation in immunecell populations associated with both innate and adaptive immune reactions. These types encompass helper T lymphocytes (HTL), cytotoxic T lymphocytes (CTL), regulatory T lymphocytes, natural killer cells, dendritic cells and various immune cell subsets. In summary, the results suggest that the newly created multi-epitope mRNA vaccine exhibits favorable attributes, offering novel insights and a conceptual foundation for potential progress in vaccine development.

## 1. Introduction

*Brucella* spp. and *Mycobacterium tuberculosis (Mtb)* spp. arecommon facultative intracellular bacteria that have the ability to be cultured *in vitro* and are known to cause zoonotic diseases on a global scale [1, 2]. The Brucella genus is predominantly comprised of 12 species, namely B.melitensis, B. abortus, B. suis, B. ovis, B. canis, B. inopinata, B. neotomae, B. microti, B. vulpis, B. ceti, B. papionis, and B. pinnipedialis. B. melitensis is the primary causative agent for the global dissemination of the disease [3]. Similarly, the Mycobacterium tuberculosis complex (MTBC) consists of seven primary phylogenetic lineages, denoted as Lineages 1–7, among which lineage 4 is notably associated with the global dissemination of the disease [4]. The clinical presentations of brucellosis closely resemble those of Tuberculosis (TB), characterized by symptoms such as fever and flu-like manifestations. Both diseases necessitate similar treatment regimens involving the administration of multiple antibiotics. Moreover, the timely identification of these conditions may present difficulties as a result of the protracted duration required for bacterial cultures and theconstrained accuracy and precision of diagnostic assays [5, 6]. Simultaneously, these chronic systemic illnesses are distinguished by the exitence of granulomas and are prevalent in diverse araes of Central and South America, Africa, and northern China [7]. However, the long-term presence of brucellosis and TB leads to notable levels of mortality and economic repercussions, primarily attributed to the emergence of drug-resistant strains in tuberculosis, such as multi-drug resistant (MDR-TB) and extensively drug-resistant (XDR-TB) strains. Research conducted by the World Health Organization (WHO) indicates that approximately 240,000 individual have died as a result of tuberculosis. The average cost related to the treatment of tuberculosis, spanning a period of 6 to 32 months, is estimated to be around $17,000 [8, 9]. Vaccination is widely recognized as a highly efficient and economical strategy for safeguarding individuals against brucellosis and tuberculosis [10, 11].

Regrettably, there is currently no available efficacious vaccine for human utilization in providing protection against Brucella spp [12]. Furthermore, at present, the sole approved vaccine for tuberculosis in humans is the Bacillus Calmette-Guerin (BCG) vaccine for Mtb in humans. However, its effectiveness in providing protection is constrained, and there are apprehensions regarding its administration to individuals with compromised immune systems and the possibility of the pathogen regressing to a more aggressive strain [13, 14]. To date, there has been a lack of success in developing a viable human vaccine targeting both *Brucella* spp. and *Mtb* spp. Consequently, it is advisable to devise an optimized vaccine that can offer robust protection to humans through a unified vaccine formulation [15].

MRNA vaccines offer several benefits including adaptability, robust immunogenicity, safety, minimal constraints, and the potential for multiple administrations [16]. Moreover, the main goal of vaccine research is to employ computational methods to identify peptides that exhibit a strong binding affinity to the surface of the target antigen [17]. The combination of outer membrane protein 25 (Omp25) and outer membrane protein 31 (Omp31) from *B*. *melitensis* exhibits efficacy in the development of vaccines capable of eliciting potent immune responses and conferring protective immunity [18]. Likewise, MPT70/Rv2875 and MPT83/Rv2873 are protective antigens with immunogenicity in Mtb-infected mice and appear to be another potential candidates for vaccine development [19]. As a result, these four proteins are being evaluated as potential target antigens for the development of mRNA vaccine formulations.

In our research, we employed an immunoinformatics methodology to examine a set of predetermined proteins, connecting the primary cellular epitopes of these proteins through designated linkers. The findings indicated that the creation of a vaccine solely utilizing epitopes posed challenges in generating a strong immune reaction. Nevertheless, the inclusion of an adjuvant in the vaccine pdemonstrated advantages by preserving the efficacy of the antigen, extending its presence in the organism, and promoting increased antibody generation [20, 21]. Therefore, it is crucial to incorporate suitable adjuvants in conjunction the primary epitope proteins to enhance the immune response to the vaccine. Through an examination of physical and chemical properties, along with the expected structure, molecular docking, and molecular dynamics simulation of the mRNA vaccine, we explored the viability of the developed mRNA vaccine as a vaccination option. This assessment will provide a theoretical basis for further research on vaccines targeting brucellosis and tuberculosis.

## 2. Methodology

### 2.1. Sequence acquisition of antigens and homology detection

The FASTA sequences and the PDB tertiary structure of Omp25 of *B*. *melitensis*, Omp31 of *B*. *melitensis*, MPT70 of strain H37Rv being of (L4) lineage 4, and MPT83 of L4 was obtained by applying the Uniprot [22] (https://www.uniprot.org/) online. Moreover, to avoid autoimmune responses, we applied the protein Basic Local Alignment Search Tool (BLASTp) of NCBI to detect whether the antigen is homologous to Homo sapiens, with its e-value set to 10^−4^ [23].

### 2.2. Prediction of transmembrane regions and signal peptides

Proteins located in the extracellular membrane play a vital role in enabling the adhesion, invasion, and long-term survival of pathogens in the human host [24]. The accuracy of TMHMM-2.0 [25] (https://services.healthtech.dtu.dk/service.php?TMHMM-2.0) is 97–98%, which is applied to the structural prediction of the protein transmembrane region to analyze whether it is located outside the membrane. In the interim, it is important to note that signal peptides are absent in native proteins and thus necessitate their exclusion during epitope analysis [26]. SignalP-6.0 (https://services.healthtech.dtu.dk/service.php?SignalP)utilizes protein language models to predict five distinct types of signal peptides and is employed for the purpose of predicting signal peptides in proteins [27].

### 2.3. Prediction of Helper T lymphocytes epitopes

When forecasting T-cell epitopes, it is crucial to take into account the diverse polymorphic characteristics of major histocompatibility complex (MHC) molecules, given the substantial variability in the distribution of human lymphocyte antigen (HLA) across different ethnic groups [28]. Therefore, the alleles HLA-DRB1*0701, HLA-DRB1*1501, and HLA-DRB1*0301 were chosen as potential predictors due to their prevalent expression in populations residing in regions where vaccines are administered [29]. Of course, as we know, helper T lymphocyte (HTL) epitopes bind primarily to MHC-II andplay a crucial role in triggering an immune response. IEDB [30] (http://tools.immuneepitope.org/) and The NetMHC-IIpan-4.0 [31] tool (https://services.healthtech.dtu.dk/service.php?NetMHCIIpan-4.0) was utilized for the prediction of Helper T Lymphocyte (HTL) epitopes. The amino acid length is 15 mer. The default threshold of NetMHC-IIpan-4.0 will remain unchanged. Following the execution of the prediction process on two servers, the top 10 epitopes will be chosen for subsequent examination.

### 2.4. Predicting epitopes for cytotoxic T lymphocytes

The predicted cytotoxic T lymphocytes (CTL) epitopes hold promise for stimulating cellular immunity and inducing the development of memory T cells [32]. Similarly, after selecting HLA-A*1101, HLA-A*0201, and HLA-A*0301 alleles, we used IEDB and NetCTLpan1.1 server [33] (https://services.healthtech.dtu.dk/service.php?NetCTLpan-1.1) for CTL epitopes prediction. Based on the aforementioned alleles, a 10-mer amino acid sequence was chosen without altering the initial parameters. The initial ten epitopes from two software scoring systems were identified, revealing a shared sequence that represents the CTL dominant epitope.

### 2.5. Predicting linear B-cell epitopes

B-cell epitopes possess the capacity to activate B cells, leading to the generation of targeted antibodies that are essential in the humoral immune response [34]. ABCpred [35] (www.imtech.res.in/raghava/abcpred/) was used to predict linear B cell (LB) epitopes. A window length of 16 was chosen to predict LB epitopes because when the software was tested by quintuple cross-validation, the overall prediction accuracy was 65.93%, the sensitivity was 67.14%, and the specificity was 64.71%, and the positive predictive value was 65.61%. Ultimately, epitopes scoring 0.9 or above were chosen as substitute LB epitopes.

### 2.6. Predicting the structural characteristics of B-cell epitopes

The vast majority of B-cell epitopes, over ninety percent, are recognized as conformational B-cell (CB) epitopes, also known as discontinuous B-cell epitopes in scholarly works [36]. ElliPro [37] (http://tools.iedb.org/ellipro/) was used to predict the CB epitopes of the target proteins. The lower limit is established at 0.5, while the upper threshold for distance (measured in angstroms) is defined as 6. We select conformational B cell epitopes within this range.

### 2.7. Identification of prominent epitopes

AllergenFP v1.0 (http://www.ddg-pharmfac.net/AllergenFP/) was used to detect whether epitopes are allergens with 88% accuracy [38]. The ToxinPred [39] server (https://webs.iiitd.edu.in/raghava/toxinpred/design.php) was used to predict whether the epitope was toxic, and the VaxiJen 2.0 server (http://www.ddg-pharmfac.net/vaxijen/VaxiJen/VaxiJen.html) was used to detect the antigenicity of the epitope with a threshold of 0.4 and an accuracy rate of approximately 89% [40]. The IFN-γ epitope server, achieving an accuracy of 82.10% with a Matthews correlation coefficient (MCC) of 0.62 on the master dataset, successfully identified IFN-γ epitope sequences within HTL epitopes, excluding those that tested negative [41].

#### 2.7.1. The molecular docking of T cell dominant epitopes with HLA alleles in a three-dimensional (3D) interaction

A molecular docking analysis was performed utilizing the HDOCK platform to evaluate the presentation of primary epitopes in relation to HLA alleles [42] platform available at http://hdock.phys.hust.edu.cn/. Prominent HLA alleles (specifically HLA-A*02:01 and HLA-DRB1*01:01) were chosen for interaction with dominant T cell epitopes. HLA-A*02:01 is the most common HLA allele in the world [43]. HLA-DRB1*01:01 exists in 95% of the population [44] Two-dimensional (2D) visualizations depicting hydrogen bonding and hydrophobic interactions were created utilizing Ligplot, whereas a three-dimensional (3D) graphical representation was generated using Pymol [45].

### 2.8. Construction of a mRNA vaccine vaccine

CTL epitopes and B cell epitopes with higher antigenicity than threshold, non-toxicity, and non-sensitization were the dominant epitopes. On this basis, the HTL epitopes were selected, and the interferon-gamma (IFN-gamma)-positive epitopes were selected as the dominant epitopes. The AAY linker was applied to connect the CTL dominant epitope, the GPGPG linker was applied to connect the HTL dominant epitope, and the KK linker was applied to connect the B-cell dominant epitope.

Human β-defensin 3 (hBD3) is a cationic antibacterial peptide with potent bactericidal activity *in vitro* [46]. It has the capacity to enhance the transmission of inflammatory signals and trigger the initiation of the adaptive immune response through antigen-presenting cells, such as dendritic cells [47]. HBD3 was chosen as an adjuvant for the vaccine. The adjuvant sequence was connected to the N-terminus of the vaccine structure using the EAAAK linker. To guarantee proper translation and expression of proteins following mRNA vaccine administration, additional components such as tissue plasminogen activator (tPA), the 5’ and 3’ Untranslated Regions (UTRs), a Kozak sequence, and a stop codon were included.

### 2.9. Characteristics of mRNA vaccines

#### 2.9.1. Physicochemical properties

By constructing, we obtained mRNA vaccines and analyzed their antigenicity and allergenicity. SOLpro [48] (http://scratch.proteomics.ics.uci.edu/) was used to predict the solubility of mRNA vaccines with 10-fold cross-validation by multiple runs with a threshold of 0.5 and an overall accuracy of 74.15% for the server. ProtParam [49] (http://web.expasy.org/protparam/) is used to predict the physicochemical properties of mRNA vaccines, that is, molecular weight, isoelectric point (PI), and the grand average of hydropathicity (GRAVY).

#### 2.9.2. Prediction of secondary and tertiary structure

Applying NetSurfP-2.0 and SOPMA (http://npsa-pbil.ibcp.fr/cgi-bin/npsa_automat.pl?page=/NPSA/npsa_sopma.html) with an accuracy of more than 80% online predicted the secondary structure of mRNA vaccines, predicting the proportion of its random curl and extended strands [50]. The prediction of tertiary structure in peptides is dependent on the availability of secondary structure data. Alphafold, a leading model known for its high accuracy, was employed to forecast the tertiary structure of peptides with the aim of improving the model’s precision [51].

#### 2.9.3. Model quality assessment of the 3D structure

Even though software tools boasting high accuracy coefficients have been utilized for protein modeling, it remains essential to conduct preliminary evaluations of the model quality before proceeding with subsequent molecular-level investigations [52]. The assessment of model accuracy is determined by the quality fraction of the model, which is evaluated synthetically through the utilization of the Z-score provided by ProSA-web [53] prediction (https://prosa.services.came.sbg.ac.at/prosa.php) and Ramachandran plot of SWISS-MODEL [54] structure assessment service prediction (https://swissmodel.expasy.org/assess).

#### 2.9.4. Molecular docking

The binding domain and molecular interaction between the receptor and mRNA vaccine were predicted by the molecular docking method. TLR4 (PDB ID: 4G8A) was selected from the Protein Data Bank (PDB) [55] (https://www.rcsb.org/) for molecular docking with MEV1 in the HDOCK server. The HDOCK server utilizes a hybrid strategy to effectively incorporate binding interface information from the PDB into the receptor and ligand structures. This approach enhances the accuracy and efficiency of integrating biological information within the system [42]. Lastly, we applied Pymol and Ligplot to perform the interaction between TLR4 and mRNA vaccine.

#### 2.9.5. Molecular dynamics simulation

The molecular dynamics (MD) simulation technique is employed to assess the stability of macromolecules within the mRNA vaccine -TLR4 complex in this study, utilizing the iMODS platform [56] platform, which employs Normal Mode Analysis (NMA) to characterize the functional movements between the molecules. This simulation also generates potential trajectories to simulate feasible motions within the system. The server conducted a predictive analysis of the molecular dynamics (MD) simulation of the mRNA vaccine -TLR4, focusing on parameters such as atomic deformability, B-factors, variance, eigenvalues, covariance map, and elastic network [57]. Additionally, MD simulations were carried out using Gromacs 2021.3 [58]. The most favorable docking configuration was immersed in a rectangular container filled with TIP3P water molecules, reaching a minimum distance of 15 Å from the protein’s perimeter [59]. Subsequently, sodium (Na+) or chloride (Cl-) ions were introduced to the protein surface to balance the overall charge of the system. Following this step, the Amber ff14SB force field was utilized for the protein in all molecular dynamics (MD) simulations [60]. Following an appropriate configuration, the initial framework underwent complete minimization utilizing a combination of steepest descent and conjugate gradient techniques. Subsequently, the system underwent a gradual annealing process from 10 K to 300 K within the canonical ensemble over a period of 0.2 nanoseconds, with a mild constraint of 15 kcal/mol/Å applied to the protein backbone atoms. A 1000 picosecond density equilibration phase was conducted under an isothermal-isobaric ensemble at a specified temperature of 300 K and pressure of 1.0 atm, employing a Langevin thermostat and Berendsen barostat with collision frequency and pressure-relaxation time parameters set at 0.002 ns and 0.001 ns, respectively [61, 62]. Following appropriate minimization and equilibration steps, a successful molecular dynamics (MD) simulation lasting 100,000 picoseconds was conducted for each system. Subsequently, the binding free energies of the molecular complexes were determined through the application of molecular mechanics-generalized-Born surface area (MMGBSA) methodology, [63, 64].

#### 2.9.6. Principal component analysis

The statistical method known as principal component analysis (PCA) was employed to examine the internal dynamics and structural alterations of biological macromolecules [65]. The GROMACS [66] 2021.3 software suite was utilized to determine the position of the Cα atom within the trajectory of the simulated system. The eigenvectors and eigenvalues derived from the diagonalized matrix signify the magnitude and orientation of collective movements, with a descending order of eigenvalues indicating that the primary principal component (PC1) corresponds to the most significant collective motion, followed by PC2 representing the second most significant motion [67].

#### 2.9.7. Immune simulation

Through the C-ImmSim server [68] (https://kraken.iac.rm.cnr.it/C-IMMSIM/), the immunogenicity of the mRNA vaccine was computed. In all simulations, a position-specific scoring matrix (PSSM) was employed, with time intervals of 1, 84, and 164 (each time step being 8 hours) [69]. Concurrently, three injections were selected to replicate the anatomical areas (bone marrow, thymus, and lymph). The simulation parameter random seed was established as 12345, with the simulation volume designated as 50, and the simulation steps configured to 1050.

#### 2.9.8. Prediction of mRNA secondary structure

The secondary structure is the fundamental characteristic of messenger RNA. In order to ensure the normal translation of mRNA, the secondary structure of the constructed mRNA vaccine was predicted [70]. Afterward, RNAfold 2.4.18 [71] (http://rna.tbi.univie.ac.at/cgi-bin/RNAWebSuite/RNAfold.cgi) was used to predict the secondary structure of the optimized RNA to ensure the translation efficiency of mRNA vaccine.

#### 2.9.9. Optimization of codons and *in silico* cloning

To efficiently express this protein in a suitable host, the amino acid sequence was transformed into a nucleotide sequence using the online software EMBOSS [72] Backtranseq(https://www.ebi.ac.uk/Tools/st/emboss_backtranseq/). Optimized the codon usage and calculated the GC content and Codon Adaptation Index (CAI) using the JAVA Codon Adaptation Tool [73] (http://www.jcat.de/). Ultimately, the optimized codon was inserted into plasmid pET-28(+) using SnapGene 6.0.2 [74], and XhoI and BamHI were selected as restriction endonuclease points.

## 3. Results

### 3.1. Sequence acquisition of antigens and homology detection

The FASTA sequence of Omp25 (Accession: Q45321), Omp31 (Accession: P0A3U4), MPT70 (Accession: P9WNF5), and MPT83 (Accession: P9WNF3) were obtained from Uniprot for further analysis. After BLASTp detection, the E-value of Omp25, Omp31, MPT70 and MPT83 were 4.6e-156, 1.3e-173, 1.7e-130 and 8.8e-153, respectively. It shows that these four proteins are identified as non-homologous compared with Homo sapiens.

### 3.2. Identifying signal peptides

**Fig 1:**
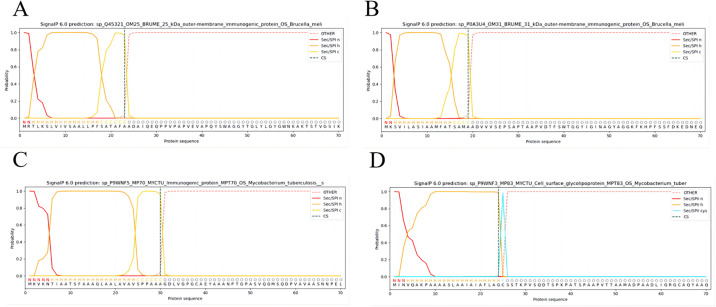
(A) Prediction of subcellular localization of protein Omp25. The pink part indicates the extracellular domain. (B) Prediction of subcellular localization of protein Omp31. (C) Prediction of subcellular localization of protein MPT70. (D) Prediction of subcellular localization of protein MPT83. (E) The N-terminal region of the signal peptide. Reported for Sec/SPI, Sec/SPII, Tat/SPI, and Tat/SPII. The signal peptide sequence of Omp25: MRTLKSLVIVSAALLPFSATAFAA. (F) The signal peptide sequence of Omp31: MKSVILASIAAMFATSAMAA. (G) Signal peptide sequence of MPT70: MKVKNTIAATSFAAAGLAALAVAVSPPAAA. (H) Signal peptide sequence of MPT83: MINVQAKPAAAASLAAIAIAFLAGC.

The findings indicated that these specific proteins are part of the outer membrane protein (Fig 1). They are located outside the cell membrane and are more useful for the design of MEVs. Further analysis, these proteins contain signal peptides that should be removed when analyzing epitopes. As shown in the figure, the signal peptides mainly include Omp25 (aa 1–24), Omp31 (aa 1–20), MPT70 (aa 1–31), and MPT83 (aa 1–25).

### 3.3. Predicting the locations of Helper T cell epitopes

Selecting the population alleles in the high incidence areas of brucellosis and TB for epitope analysis, we selected the top ten epitopes in IEDB and NetMHCIIpan-4.0. Then, find the same sequence as a candidate epitope in a common allele. Finally, 18 HTL epitopes were obtained in Omp25,18 HTL epitopes were obtained in Omp31,13 HTL epitopes were obtained in MPT70, and 12 HTL epitopes were obtained in MPT83.

### 3.4. Predicting the locations of cytotoxicr T lymphocytes

In the same way, we obtained the top ten epitopes in the IEDB and NetCTLpan1-1 software and identified the same sequence as a candidate epitope in the common allele. Finally, 11 HTL epitopes were obtained in Omp25,14 HTL epitopes in Omp31,16 HTL epitopes in MPT70, and 14 HTL epitopes in MPT83.

### 3.5. Predicting the locations of linear B-cell

By setting the software’s parameters as the default, we removed the epitopes that scored lower than the parameters. After screening, four LB epitopes were obtained in Omp25, two LB epitopes were obtained in Omp31, two LB epitopes were obtained in MPT70, and one LB epitope was obtained in MPT83.

### 3.6. Predicting the locations of conformational B-cell epitopes

**Fig 2:**
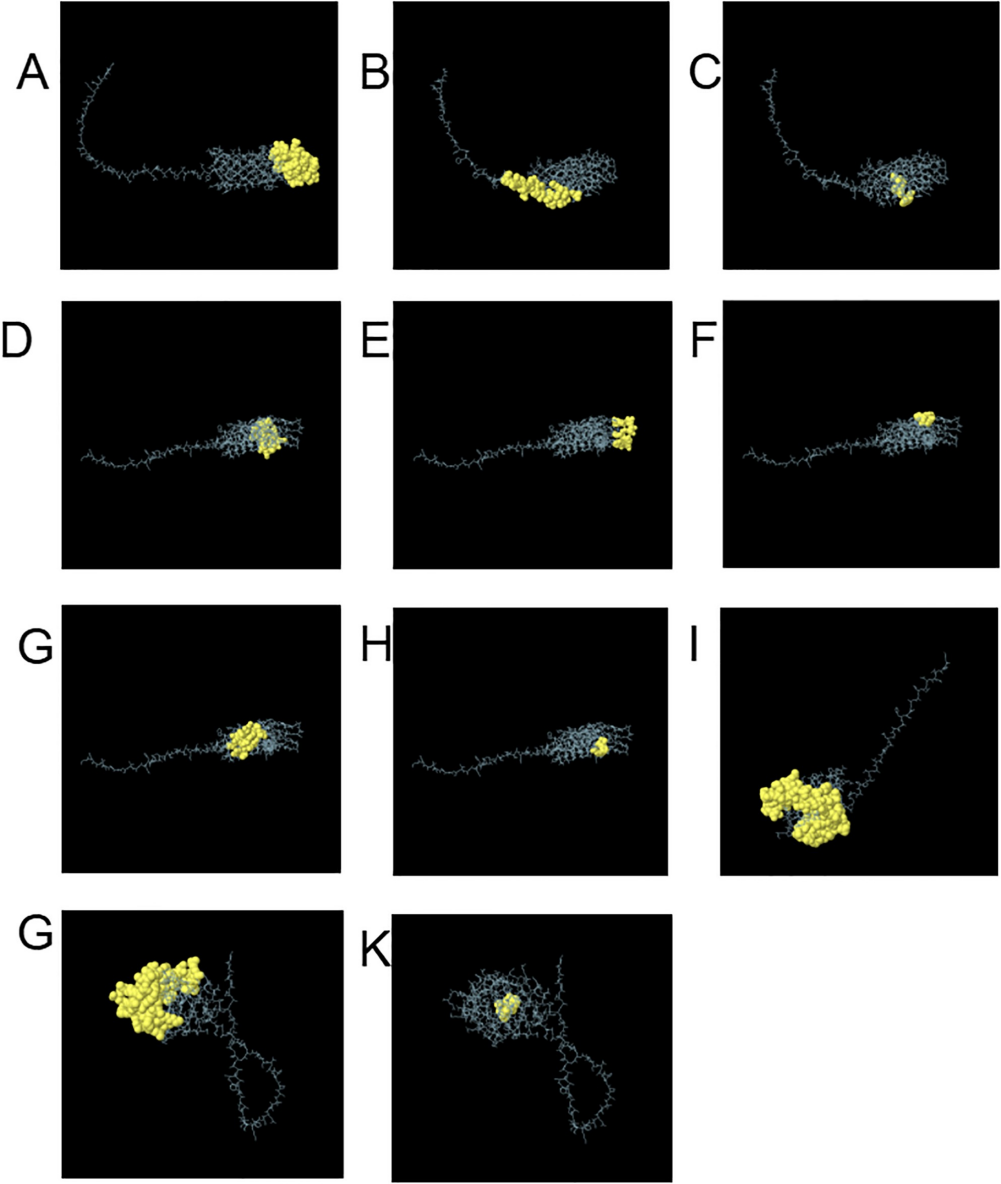
Conformational B cell epitope 3D map. (A-C)OMP25 protein B cell conformational epitope.(D-H)OMP30 protein B cell conformational epitope.(I)MPT70protein B cell conformational epitope.(G-K)MPT83protein B cell conformational epitope.

Following predictive analysis, Omp25 yielded three B-cell conformational epitopes, while Omp31 revealed five such epitopes. Additionally, MPT70 displayed one B-cell conformational epitope, and MPT83 exhibited two B-cell conformational epitopes, as outlined in Table 1 and using Ellipro to demonstrate the 3D structure of conformational B cell epitopes (Fig 2).

**Table 1:** List of the selected CB epitopes an MEV design.

residues		number of residues	Score
Omp25	A:K60, A:K62, A:T63, A:S64, A:T65, A:V66, A:G67, A:S68, A:K70, A:P71, A:S105, A:K106, A:D107, A:G108, A:L109, A:K144, A:L145, A:N146, A:N147, A:G148, A:L149, A:D150, A:D151, A:E152, A:S153, A:K154, A:G185, A:N186, A:K187, A:N188, A:Y189, A:D190, A:L191, A:A192, A:G193, A:T194, A:T195, A:V196, A:R197, A:N198, A:K199, A:L200, A:D201	43	0.682
	A:Q30, A:P31, A:P32, A:V33, A:P34, A:A35, A:P36, A:V37, A:E38, A:V39, A:A40, A:P41, A:Q42, A:Y43, A:L52, A:L127, A:N128, A:P129, A:V130, A:A167, A:L169, A:T170, A:D171, A:N172, A:I173, A:G208, A:I209, A:G210, A:Y211, A:K212	30	0.624
	A:Y56, A:W58, A:W74, A:Q203	4	0.502
OMP31	A:K55, A:K57, A:P59, A:F60, A:S61, A:S62, A:F63, A:D64, A:K65, A:E66, A:D67, A:N68, A:E69, A:Q70, A:V71, A:S72, A:G73, A:S74, A:D76, A:T78, A:K212, A:N214, A:L215, A:V216, A:D217, A:V218, A:D219, A:N220, A:S221, A:F222, A:E224	31	0.788
	A:S116, A:A117, A:G118, A:A119, A:S120, A:G121, A:L122, A:E123, A:K125, A:G168, A:D169, A:D170, A:A171, A:S172, A:A173, A:L174, A:H175	17	0.725
	A:E131, A:W132, A:K160, A:S178, A:D179, A:K180, A:T181	7	0.624
	A:F38, A:I45, A:G46, A:I47, A:G84, A:G85, A:V86, A:R147, A:Y193, A:I195, A:N196, A:N197, A:N198, A:W199, A:G235, A:L236, A:N237, A:Y238, A:K239	19	0.552
	A:Y51, A:F82, A:S110, A:V111	4	0.521
MPT70	A:V34, A:G35, A:P36, A:G37, A:P59, A:A75, A:S78, A:G79, A:Q80, A:L81, A:N82, A:P83, A:Q84, A:V85, A:N86, A:L87, A:V88, A:D89, A:T90, A:N92, A:S93, A:G94, A:Q95, A:T102, A:A104, A:S107, A:K108, A:L109, A:P110, A:A111, A:S112, A:T113, A:I114, A:D115, A:E116, A:L117, A:K118, A:T119, A:N120, A:S121, A:S122, A:A134, A:G135, A:Q136, A:T137, A:S138, A:P139, A:A140, A:N141, A:V142, A:V143, A:G144, A:T145, A:R146, A:Q147, A:T148, A:G151, A:A152, A:S153, A:T155, A:V156, A:T157, A:G158, A:Q159, A:G160, A:N161, A:S162, A:L163, A:K164, A:V165, A:G166, A:N167, A:A168, A:D169, A:T177, A:A178, A:N179, A:D186	78	0.655
MPT83	A:I60, A:G61, A:R62, A:S104, A:G105, A:K106, A:L107, A:N108, A:P109, A:D110, A:V111, A:N112, A:L135, A:P136, A:A137, A:A138, A:T139, A:I140, A:D141, A:Q142, A:L143, A:K144, A:T145, A:D146, A:A147, A:K148, A:L149, A:S151, A:S152, A:I168, A:D169, A:G170, A:T171, A:H172, A:Q173, A:T174, A:L175, A:Q176, A:G177, A:A178, A:D179, A:T181, A:V182, A:I183, A:G184, A:A185, A:R186, A:D187, A:D188, A:L189, A:M190, A:V191, A:N192, A:N193, A:A194, A:G195, A:M216, A:P217, A:P218, A:A219, A:Q220	61	0.607
	A:A163, A:S164, A:P165, A:S166, A:R167	5	0.509

### 3.7. Selection of dominant epitopes

**Fig 3:**
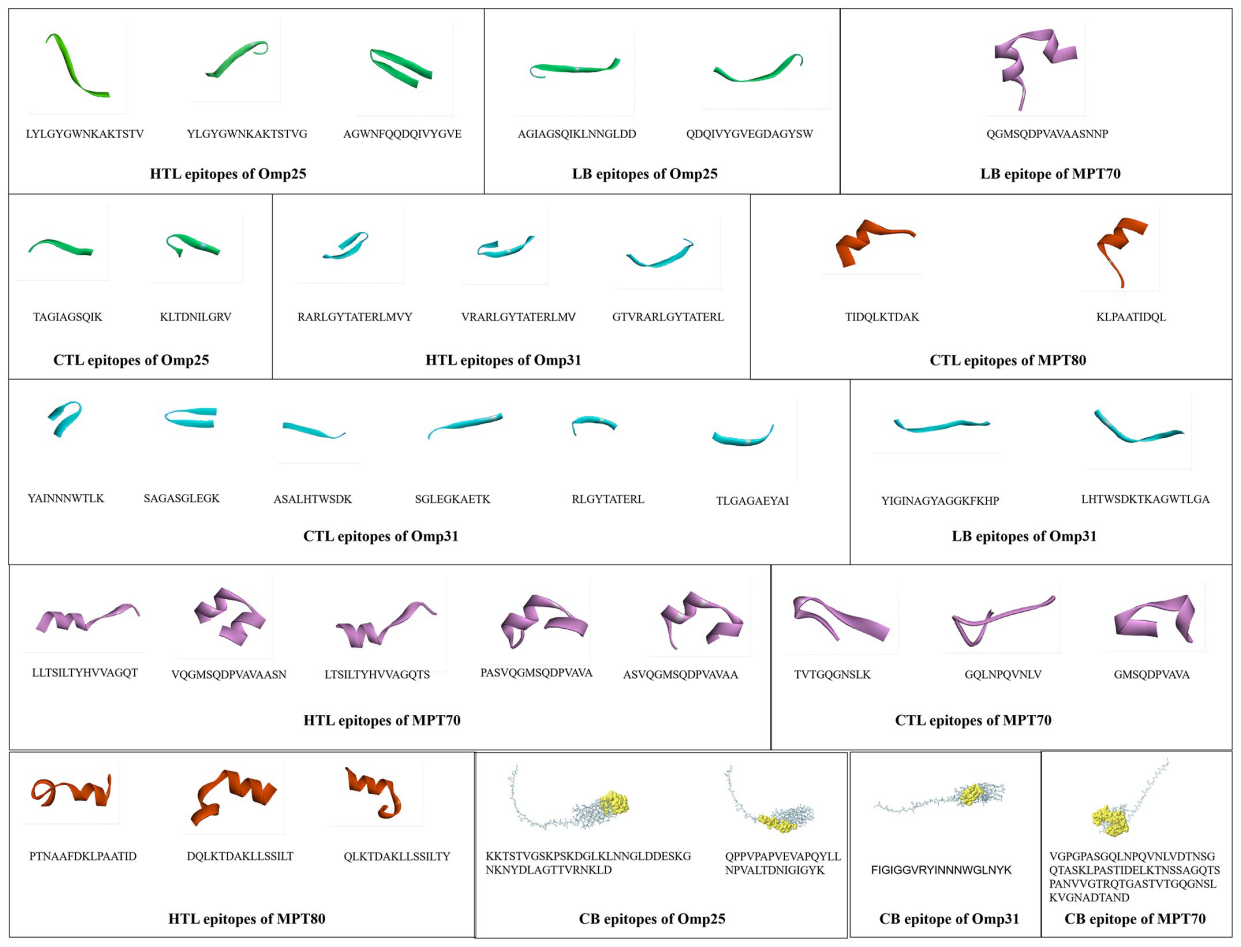
The final HTL epitopes, CTL epitopes, LB epitopes, and CB epitopes were selected for MEV design. As illustrated in the figure, “green” stands for the T-cell epitope of Omp25, “blue” stands for the T-cell epitope of Omp31, “purple” stands for the T-cell epitope of MPT70, and “red” stands for the T-cell epitope of MPT83.

After screening, three HTL epitopes, two CTL epitopes, two LB epitopes, and two CB epitopes were obtained in Omp25. In the study, a total of three helper T lymphocyte (HTL) epitopes, six cytotoxic T lymphocyte (CTL) epitopes, two B lymphocyte (LB) epitopes, and one B cell (CB) epitope were identified from Omp31. Additionally, five HTL epitopes, three CTL epitopes, one LB epitope, and one CB epitope were identified in MPT70. Lastly, three HTL epitopes, two CTL epitopes, no LB epitopes, and no CB epitopes were identified in MPT83, as illustrated in Fig 3.

#### 3.7.1. Molecular docking analysis of the three-dimensional (3D) interaction between HLA alleles and dominant T cell epitopes

**Fig 4:**
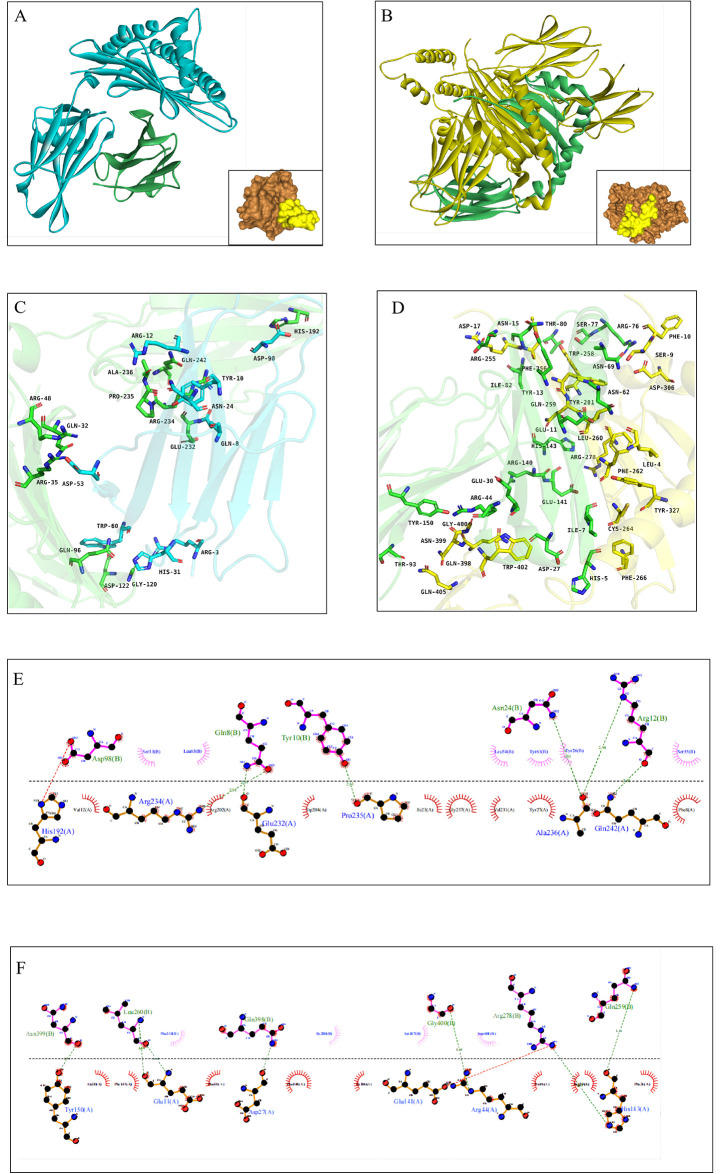
(A) Representative lateral views of HLA-A*02:01 interacting with CTL epitopes. (B) Representative lateral views of HLA-DRB1*01:01 interacting with HTL epitopes. (C) The interaction interface residues between HLA-A*02:01 and CTL epitopes were analyzed by PyMol. (D) The interaction interface residues between HLA-DRB1*01:01 and HTL epitopes were analyzed by PyMol. (E) The interaction interface residues between HLA-A*02:01 and CTL epitopes were analyzed by Ligplot. (F) The interaction interface residues between HLA-DRB1*01:01 and HTL epitopes were analyzed by Ligplot.

Following the docking process (Fig 4), the optimal docking configuration of the complex was selected for further examination. The docking scores for the interaction between HLA-A*02:01 and CTL epitopes were recorded as -175.94, with a ligand RMSD of 51.52 Å. In contrast, the docking score for the interaction between HLA-DRB1*01:01 and HTL epitopes was -210.65, accompanied by a ligand RMSD of 189.07 Å. In the 3D interaction interface, the predominant interactions between the two complexes are attributed to ionic bonds and hydrogen bonds. To illustrate the docking forces, the 2D interaction interface is employed, where the ionic bond is denoted by a red dotted line and the hydrogen bond is represented by a green dotted line.

### 3.8. Construction of a mRNA vaccine vaccine

**Fig 5:**
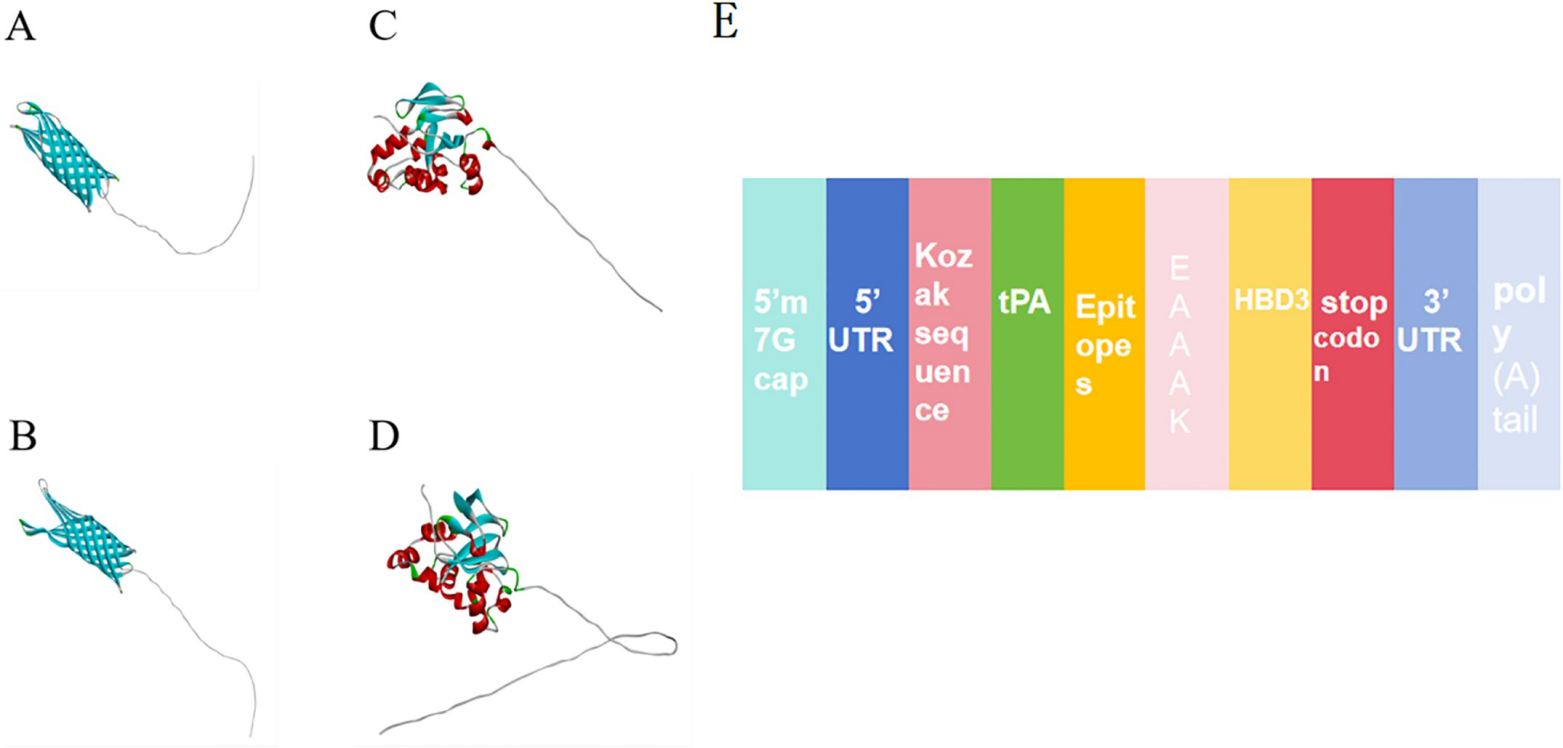
(A) The 3D structure of Omp25. (B) The 3D structure of Omp31. (C) The 3D structure of MPT70. (D) The 3D structure of MPT83. (E) The final structure diagram of the mRNA vaccines. Multi-epitope peptides(deep yellow), EAAAK(Pale pink), HBDC(faint yellow)are adjuvants.

After constructing mRNA vaccine (Fig 5), we combined adjuvant, Human β-defensin 3 (HBD3) sequence, CTL epitopes, HTL epitopes, and B cell epitopes. These peptide segment contains 14 HTL epitopes, 13 CTL epitopes, 5 LB epitopes, and 4 CB epitopes. The components of the vaccine from the N-terminus to the C-terminus are as follows. 5’m7GCap- 5’UTR—Kozak sequence-tPA (Signal peptide)-Epitopes-EAAAK-HBF3-stop codon-3’UTR-poly(A)tail.

### 3.9. Characteristics of MEVs

#### 3.9.1. Physicochemical properties of mRNA vaccine

It can be seen that our target mRNA vaccine has good physicochemical properties and is a promising candidate for vaccine design (Table 2). The mRNA vaccine consists of 790 amino acids with a molecular weight of 81043.77 kDa, a theoretical pI of 9.68, and a molecular formula of C_3588_H_5716_N_1014_O_1098_S_13_. The half-life of mRNA vaccine in *E*. *coli* is greater than 10 hours. In addition, its instability index is 21.44, the aliphatic index is 73.86, and the hydrophilic index is -0.352. After analysis, It is a good candidate for vaccines against brucellosis and TB.

**Table 2:** Physicochemical properties of the vaccine peptide.

Vaccine/adjuvant	Antigenicity	Allergenicity	Solubility	Number of amino acids	Molecular Weight	Theoretical pI	Instability Index	GRAVY
V/hBD3	0.9506	Non	0.93	790	81043.77	9.68	21.44	-0.352

#### 3.9.2. Predicting the arrangement of secondary and tertiary structures

**Fig 6:**
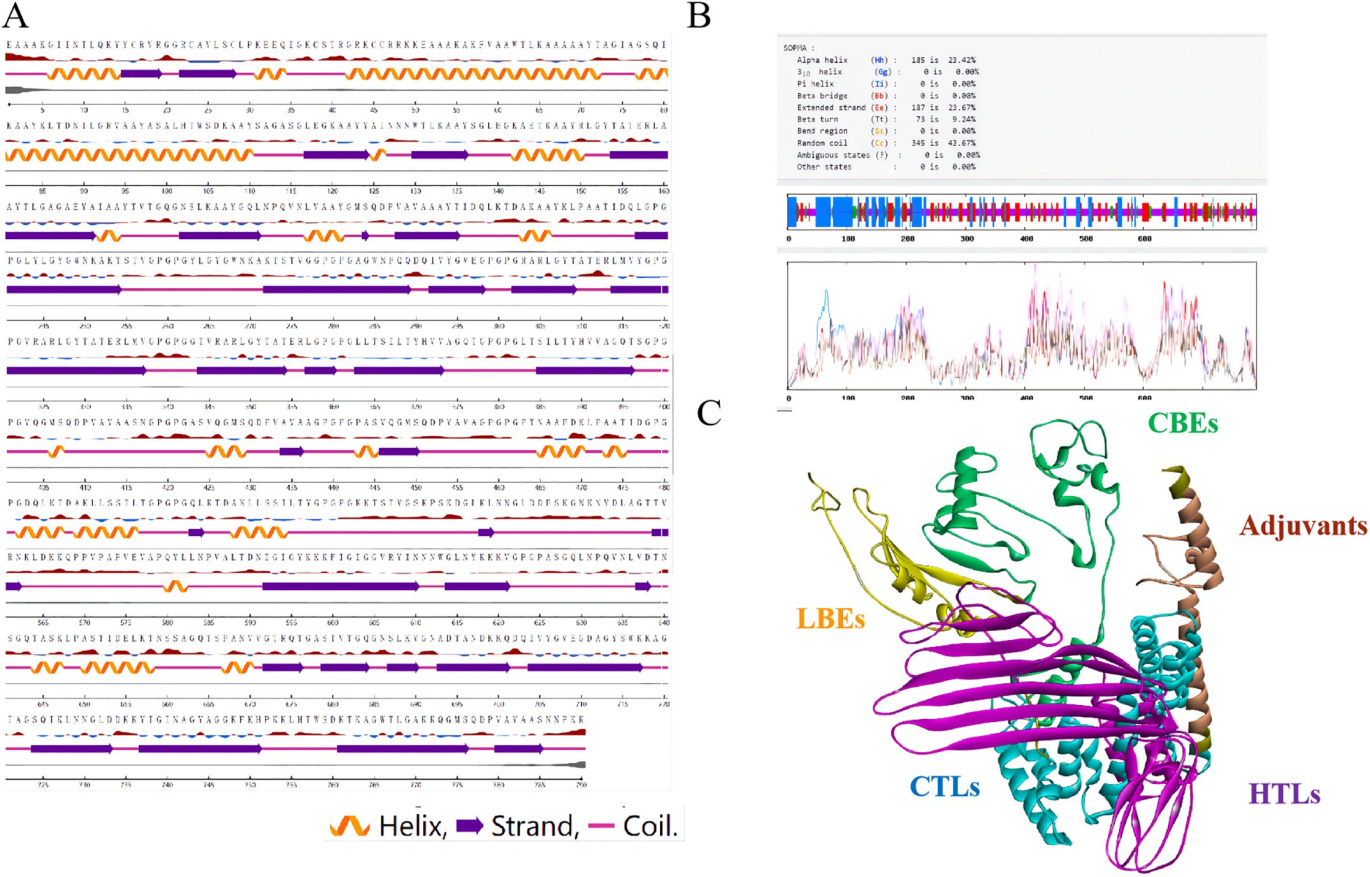
(A) The secondary structure of the mRNA vaccine was predicted by NetSurfP-2.0. With a threshold of 25%, red upward elevation implies residue exposure, whereas sky blue denotes buried residue. As illustrated in the figure, “orange” stands for the alpha-helix, “blue” stands for the β-fold, and “purple” stands for the random curl. Thereinto, the disorder is represented as a bloated black line, with the thickness of the line equaling the probability of disordered residue. (B) The secondary structure of the mRNA vaccine was predicted by SOPMA. (C) The tertiary structure of the mRNA vaccine was predicted by AlphaFold.

In the anticipated secondary configuration of the mRNA vaccine, approximately 43.67% is classified as a random coil, while around 23.67% is identified as extended strands. To better understand the three-dimensional arrangement of the mRNA vaccine, we conducted a prediction of its tertiary structure. The findings indicate that the percentage of random curl and extended strands in the tertiary form of mRNA vaccine is similar to that in the secondary structure (Fig 6). A comprehensive analysis of both shows that our prediction model is reasonable.

#### 3.9.3. Assessing the quality of the 3D structure model

**Fig 7:**
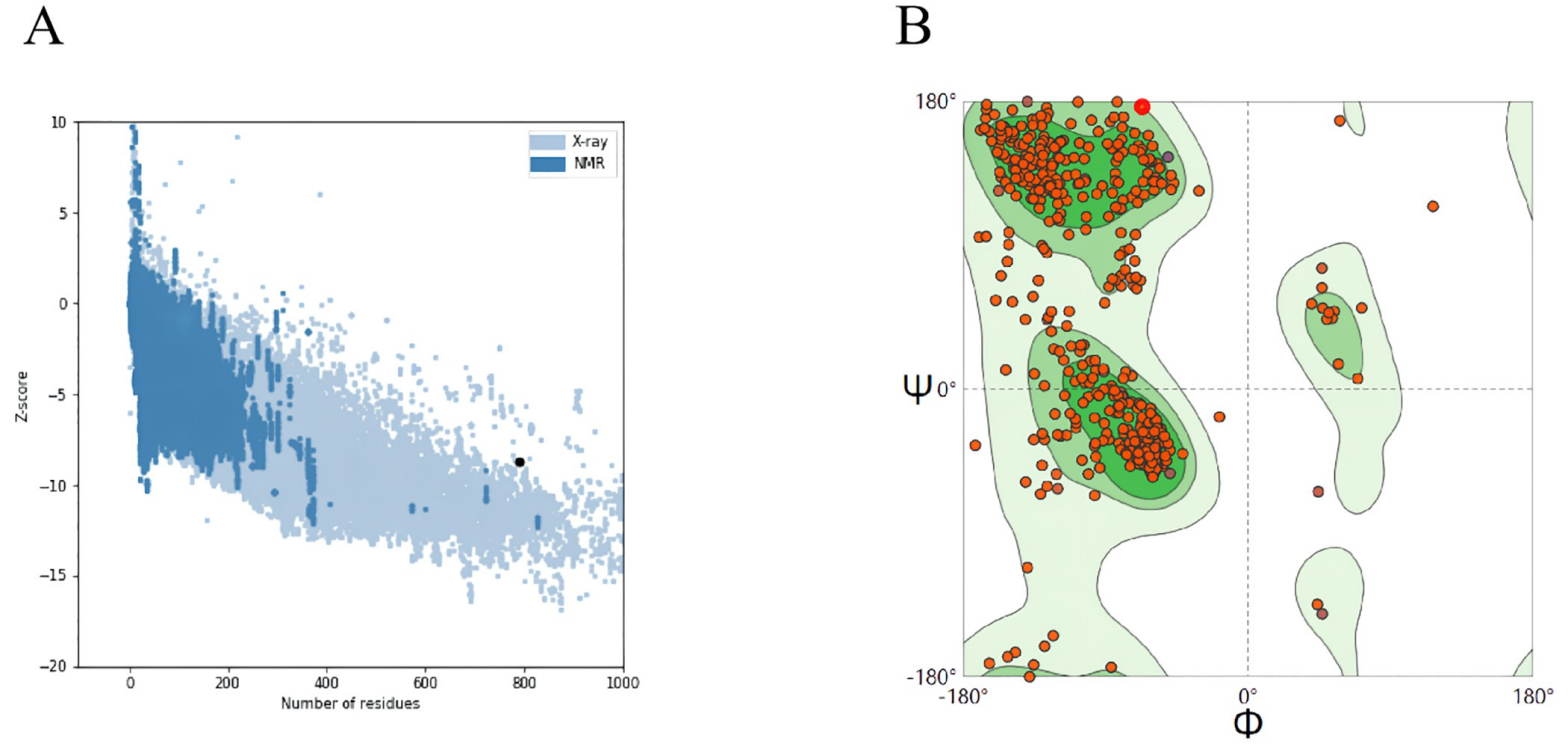
(A) Z score of the model of the mRNA vaccine model developed by ProSA-web. (B) Ramachandran plots of the mRNA vaccine. As illustrated in the figure, “dark green” stands for the most favored regions, “green” stands for the additional allowed regions, “light green” stands for the generously allowed regions, and “white” stands for the disallowed regions.

Following the assessment of the mRNA vaccine’s three-level structure, the model’s Z-score is -8.72. A lower Z-score indicates higher model quality and reliability. Additionally, to confirm the quality assessment, we can observe in the Ramachandran plot that the model contains 93.02% favorable regions, 6.22% generously allowed regions, and 0.76% outlier regions. The analysis results, along with two prediction tools, indicated that the projected tertiary structure model was of high quality and had strong confidence in its accuracy. This model can be utilized for additional analysis (Fig 7).

#### 3.9.4. Molecular docking

**Fig 8:**
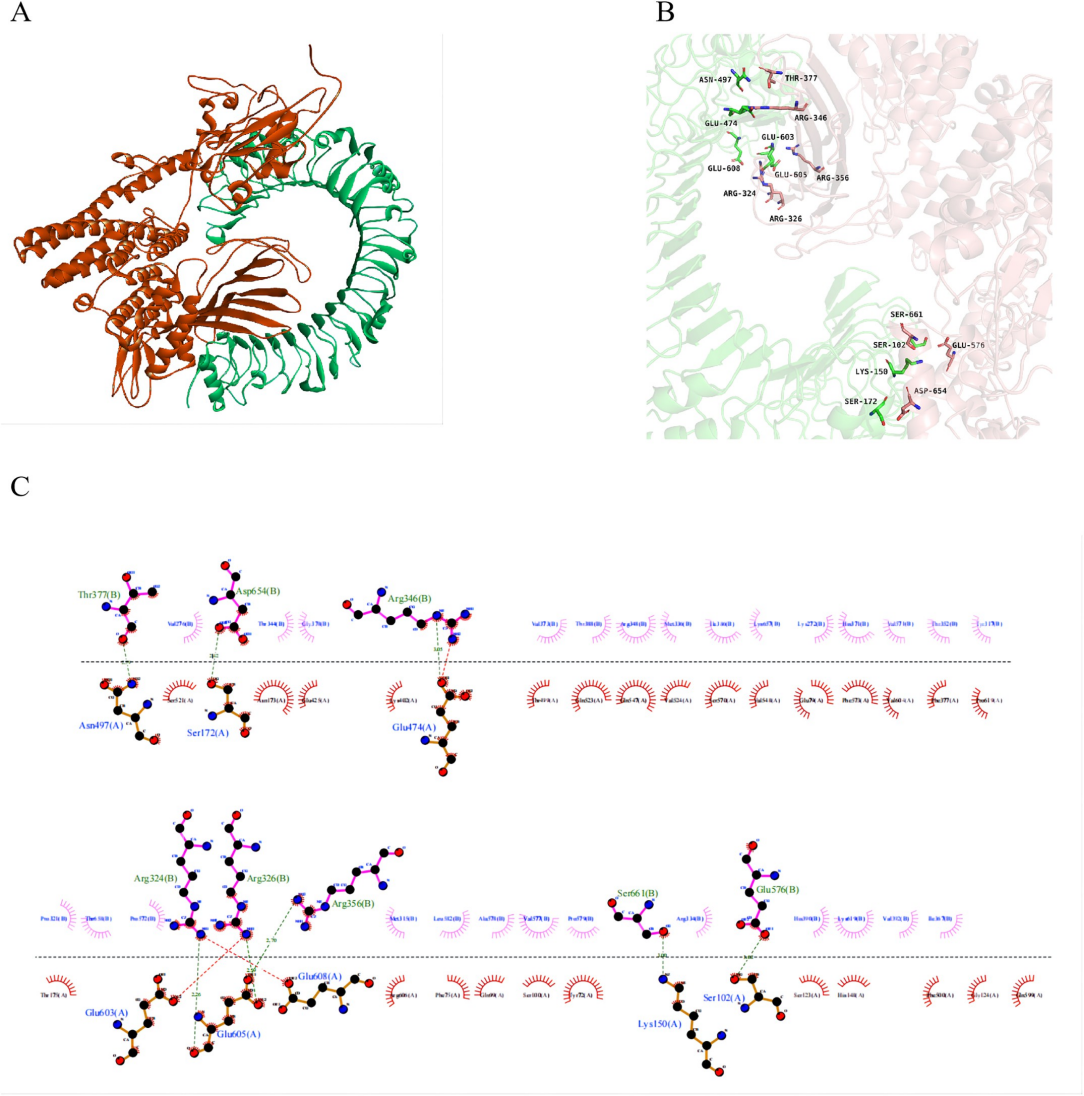
(A) The docking model of mRNA vaccine -TLR4 predicted by HDOCK. (B) The interaction interface residues between mRNA vaccine and TLR4 were analyzed by PyMol. (C) The interaction interface residues between mRNA vaccine and TLR4 were analyzed by Ligplot.

The docking scores serve as a measure of the stability of the complexes, where lower scores correspond to greater stability [75]. The best docking complex was chosen for examination. The findings revealed that the top complex of mRNA vaccine-TLR4 had a docking score of -352.36 and a ligand RMSD of 109.48 Å. We demonstrate the interaction between mRNA vaccine and TLR4 at the 2D and 3D structural levels. Thereinto, the force mainly includes hydrogen bonds and Van der Waals forces (Fig 8).

#### 3.9.5. Molecular dynamics simulation

**Fig 9:**
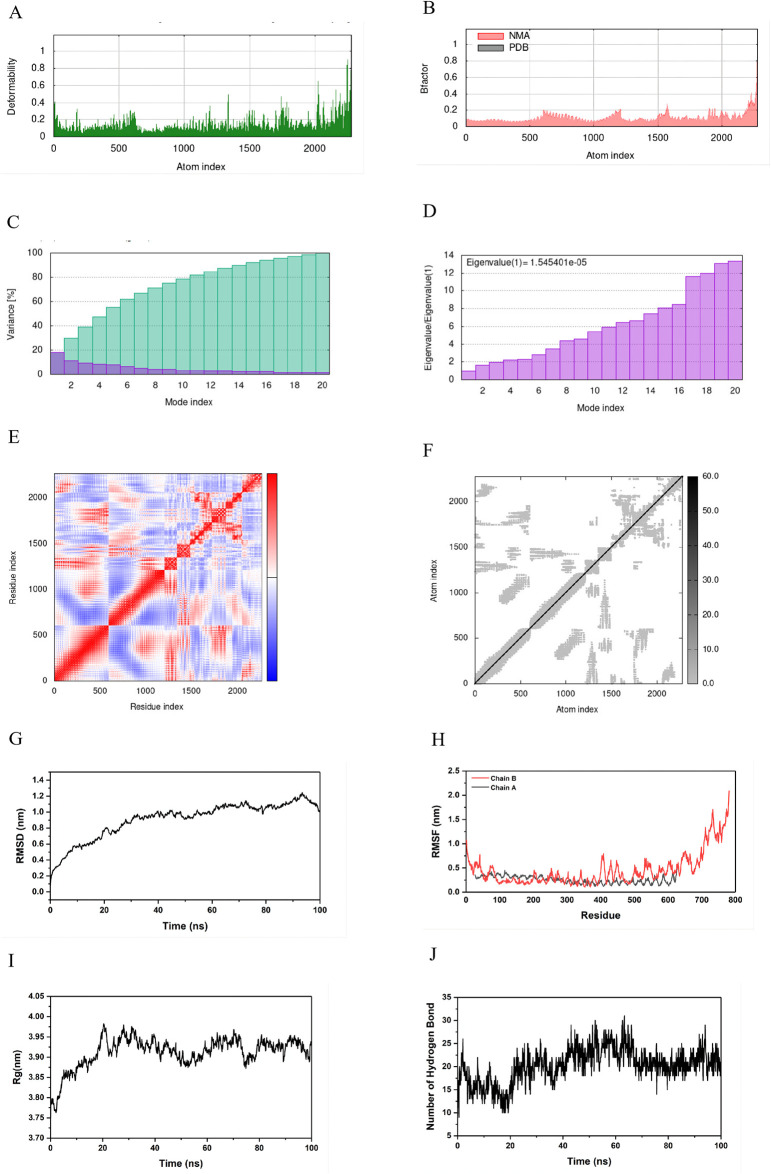
(A) The deformability plot represents the complexes formed by the combination of mRNA vaccine with TLR4. (B) The B-factor value plot represents the complexes formed by the combination of mRNA vaccine with TLR4. (C) The variance plot represents the complexes formed by the combination of mRNA vaccine with TLR4. (D) The eigenvalue plot represents the complexes formed by the combination of mRNA vaccine with TLR4. (E) The covariance matrix represents the complexes formed by the combination of mRNA vaccine with TLR4. As illustrated in the figure, “red” stands for the correlated motions, “white” stands for the uncorrelated motions, and “blue” stands for the anti-correlated motions. (F) The cross-correlation matrix diagram represents the complexes formed by the combination of mRNA vaccine with TLR4. (G) Root-mean-square deviation (RMSD) of the mRNA vaccine -TLR4. RMSD over the entire simulation, where the ordinate is the value of RMSD, and the abscissa is time (ns). (H) RMSF values of the mRNA vaccine -TLR4 over the entire simulation, where the ordinate is RMSF, and the abscissa is residue. (I) The radius of gyration (Rg) over the entire simulation, where the ordinate is Rg, and the abscissa is time (ns). (F) The number of total hydrogen bonds versus simulation time.

The examination revealed minimal distortion in the residues of the complex as depicted in the deformability plot (Fig 9), with the B-factor values determined through NMA exhibiting less deviation compared to the values of the mRNA vaccine -TLR4. Concurrently, the eigenvalue of the complex system was 1.55e-05 and exhibited a gradual increase in each mode over the course of the dynamics. A lower eigenvalue indicates greater ease of deformation and heightened instability in the composite structure [76]. The variance plot illustrated a progressive decline in the individual variance across each subsequent mode. The covariance and cross-correlation matrices offer insights into the correlation among atomic movements and the magnitude of these movements [77]. In addition to other factors, the correlations of atomic movements primarily consist of correlated (indicated by the red region), uncorrelated (depicted by the white region), and anti-correlated (represented by the blue region) motions. In the covariance matrix illustration, significant correlations are observed between the atoms of the two docking complexes. The comprehensive evaluation of iMODS indicates increased rigidity and reduced deformation in the internal coordinates of the complex throughout the dynamics, thereby supporting the stability of the interface between the mRNA vaccine -TLR4 complex.

Moreover, in order to assess the structural integrity of the mRNA vaccine—TLR4 complex, molecular dynamics simulations lasting 100 nanoseconds were conducted. The complex initially displayed a significant rise in the Root Mean Square Deviation (RMSD) value at the onset of the simulation. Subsequently, structural fluctuations within the complex reached a state of equilibrium after 39 nanoseconds, maintaining an RMSD value of approximately 0.4 nanometers for the duration of the remaining simulation, with an average RMSD of 1 nanometer. Analysis of the RMSD data indicated that the interaction between the mRNA vaccine and TLR4 led to the stabilization of the complex’s structure. A lower RMSD value corresponded to a more stable configuration of the complex [78]. The examination of the root mean square fluctuation (RMSF) of the complex serves as an indicator of the stability of the interaction between the mRNA vaccine and TLR4 throughout molecular dynamics (MD) simulations. RMSF values were computed for the backbone atoms of the residues within the complex, revealing that the two chains of the complex exhibited lower RMSF values (&lt;1 nm) for residues 0–700, while residues beyond position 700 displayed higher RMSF values (&gt;1 nm). The compactness of the mRNA vaccine-TLR4 complex was assessed by analyzing the radius of gyration (Rg) data, which demonstrated that the gyration of the backbone atoms in the complex structure was initially unstable before 20 ns, fluctuating between 3.73 nm and 3.93 nm. Subsequently, post 20 ns, the gyration of the structure began to stabilize at around 3.90 nm for the remainder of the simulation period. Evaluation of the hydrogen bonds formed between the mRNA vaccine and TLR4 within the complex elucidated the interface stability during MD simulations. The analysis revealed that an average of 20 hydrogen bonds were consistently present at the interface from 20 to 100 ns, signifying enhanced stabilization of the interaction between the mRNA vaccine and TLR4. Lastly, the average binding energy of the complex was determined to be -499.9713 kJ mol-1 utilizing the MMGBSA tool within the GROMACS software suite.

#### 3.9.6. Principal component analysis

**Fig 10:**
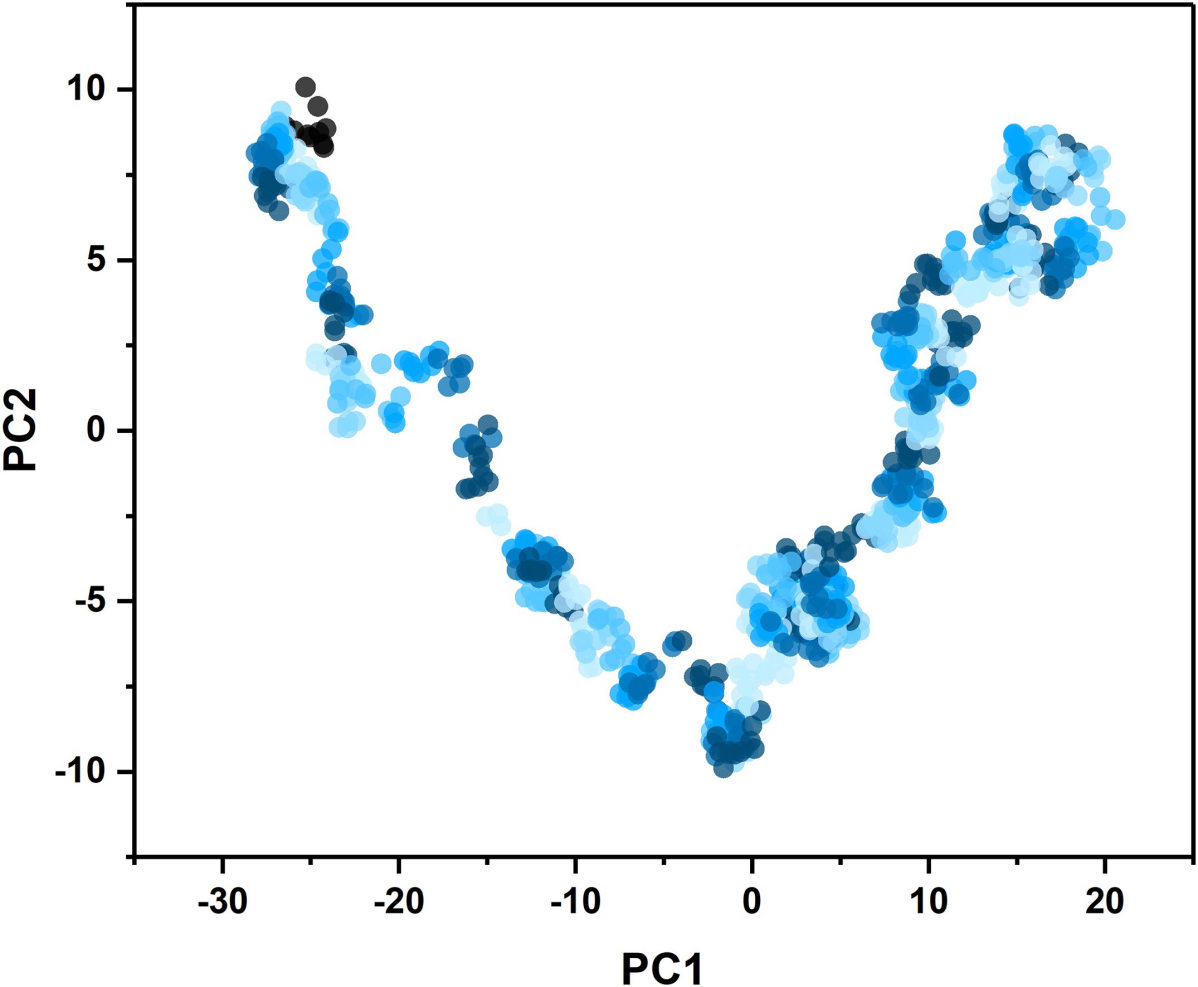
Projection of trajectories into PC1 and PC2 for the mRNA vaccine -TLR4 complex.

Principal Component Analysis (PCA) was conducted on the acquired 100 nanosecond simulation trajectories of the mRNA vaccine—TLR4 complex system. PCA, an unsupervised technique for reducing dimensionality, was utilized to map the data onto a two-dimensional space defined by the principal component 1 (PC1) and principal component 2 (PC2) coordinates. The PC1 values for the mRNA vaccine—TLR4 complex were observed to be distributed within the range of -30 to -20 nanometers, while the PC2 values were distributed within the range of -10 to 10 nanometers (Fig 10). The limited fluctuation range in the distribution of conformations suggests a high degree of stability in the docking complexes formed by the mRNA vaccine and TLR4.

#### 3.9.7. Immune simulation

**Fig 11:**
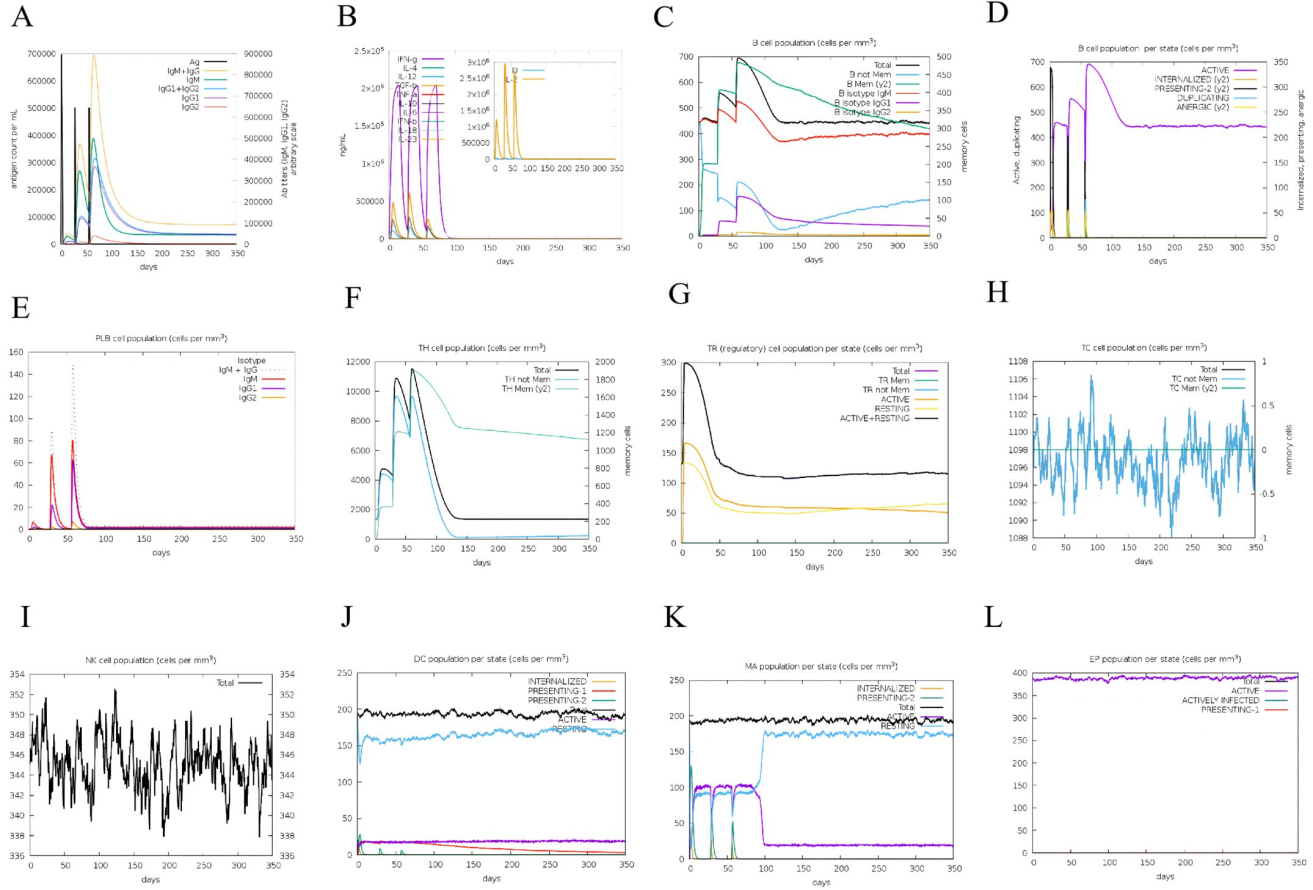
(A) The level of immunoglobulins after vaccination. As illustrated in the figure, “yellow” stands for the IgM+IgG, “green” stands for the IgM, “blue” stands for the IgG1+IgG2, “purple” stands for the IgG1, and “red” stands for the IgG2. (B) The concentration of cytokines and interleukins after vaccination. As illustrated in the figure, “D” stands for the danger signal. (C) The level of B-cell populations after vaccination. As illustrated in the figure, “black” stands for the total number of B cells, and “green” stands for the memory B cell. (D) B lymphocytes population per entity-state (i.e., showing counts for active, presenting on class-II, internalized the ag, duplicating, and anergic. (E) The level of plasma B lymphocytes after vaccination. (F) The level of CD4 T-helper lymphocytes after vaccination. (G) The level of CD4 T-regulatory lymphocytes after vaccination. Both total, memory, and per entity-state counts are plotted here. (H) The level of CD8 T-cytotoxic lymphocytes after vaccination. (I) The level of natural killer cells after vaccination. (J) The level of dendritic cells after vaccination. The curves show the total number broken down to active, resting, internalized, and presenting the ag. (K) The level of macrophages after vaccination. (L) The level of epithelial cells after vaccination.

By immune simulation for mRNA vaccine (Fig 11), we found that after vaccine injection, it could cause fluctuations in the associated immune cells of both innate and adaptive immune responses. These include HTL, CTL, T-regulatory lymphocytes, natural killer cells, dendritic cells, and other immune cells. With the injection of three doses of vaccine, immunoglobulins (IgM, IgG1, IgG2, IgG1+IgG2, and IgM+IgG) showed an increasing trend. And after the last vaccine injection, the level of immunoglobulin reached its peak (Fig 11A). The cytokines (IFN-γ, TGF-β, IL-10, and IL-12) in the host were also increased after three doses of the vaccine (Fig 11B). Thereinto, the increase of IFN-γ was predominant. However, the risk factors did not fluctuate significantly during this process. The general B-cell presence is gradually rising, mainly based on the rise of memory B cells (Fig 11C and 11D). Plasma cells (Fig 10E) also showed a gradually rising trend, and the produced antibodies are mainly IgM and IgG1. Similarly, TH cells (Fig 11F) have also gradually risen, mainly based on the rise of memory T cells. T-regulatory lymphocytes (Fig 11G) gradually showed a downward trend after rising to the highest point. CTL (Fig 11H), natural killer cells (Fig 11I), dendritic cells (Fig 11J), macrophages (Fig 11K), and epithelial cells (Fig 11L) are in a relatively stable dynamic state. Specifically, dendritic cells have the ability to display antigenic peptides on MHC class-I and class-II molecules, facilitating the presentation of antigens to T-cells [79]. This comparative analysis highlights the mRNA vaccine’s effectiveness in generating a strong immune response against B. melitensis and Mtb H37Rv (L4 strain), indicating its potential as a powerful vaccine.

#### 3.9.8. Predicting the secondary structure of mRNA vaccines

**Fig 12:**
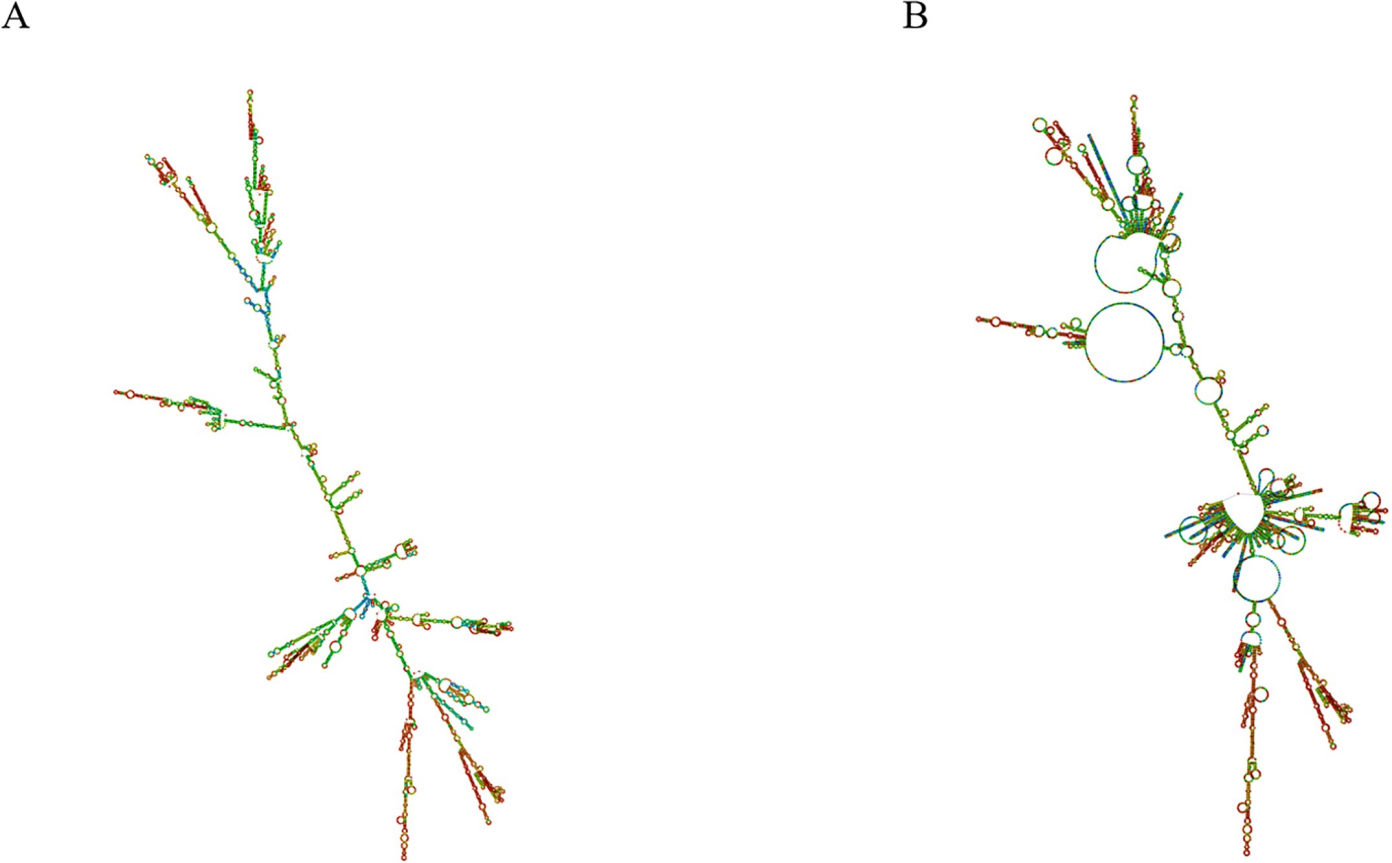
RNA secondary structure. (A) The optimal secondary structure of mRNA vaccine has the minimum free energy. (B) The centroid structure of mRNA vaccine.

Following codon optimization, the Codon Adaptation Index (CAI) value reaches 1.0, while the guanine-cytosine (GC) content is measured at 53.21%. To further evaluate the optimized codon, we predicted the secondary structure of its RNA. The minimum free energy of RNA is -807.30 kcal/mol. The more negative the free energy, that is, the greater the absolute value and the more stable the secondary structure formed [80]. The results show that the optimized codon can be analyzed in a reasonable range of parameters (Fig 12).

#### 3.9.9. Optimization of codons and *in silico* cloning

**Fig 13:**
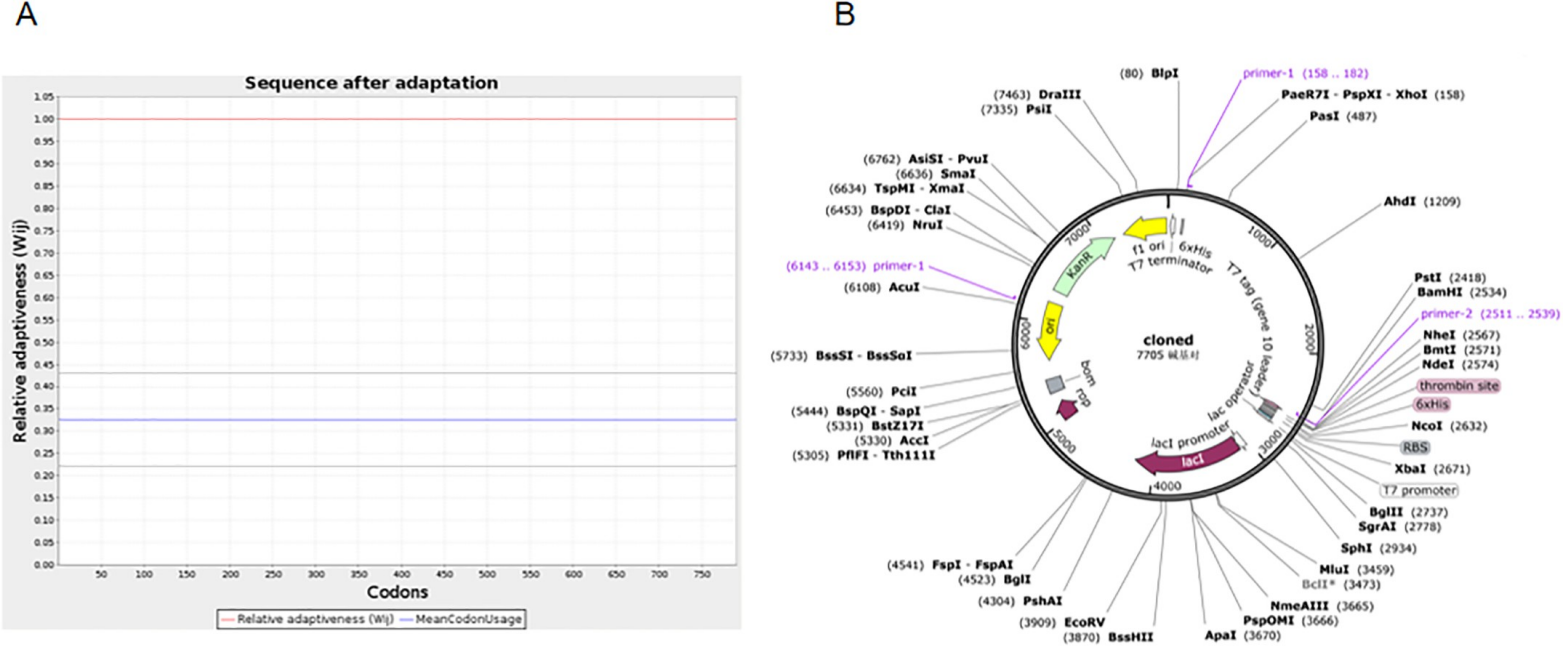
(A) The calculation of the codon adaptation index. (B) *In silico* cloning of the mRNA vaccine optimized codons (purple) into pET-28a (+) expression vector between XhoI and BamHI restriction sites.

Following codon optimization, a DNA sequence comprising 2370 amino acids was generated. Subsequently, the optimized codon was introduced into the pET-28a (+) vector for the purpose of cloning. Ultimately, a recombinant plasmid of 7705 base pairs was produced, laying the groundwork for the development of a potential vaccine.(Fig 13).

## 4. Discussion

Brucellosis and tuberculosis are notable chronic infections that are widespread in many regions worldwide, especially in agriculturally disadvantaged areas [81]. The persistent nature of these diseases raises societal expenses and reduces quality of life. As antibiotic resistance grows, there is a pressing requirement for strategies to prevent and manage these conditions [82]. Ideally, developing a single vaccine formulation to confer protection against both bacteria would prevent their occurrence at the source [83]. So far, however, there is no combination vaccine against both bacteria. Polyvalent vaccine research is a promising approach to decrease the number of immunizations needed and lower vaccine costs [84].

Immunoinformatics is viewed as a promising, expedient, and dependable approach for identifying T and B cell epitopes, surpassing the efficacy of individual antigens or traditional inactive pathogen vaccines [41, 85], Creating a new vaccine using immunoinformatics tools shows potential in stopping the spread of infectious diseases [86]. Hence, we proceeded with analyzing the epitopes of B. melitensis, and utilized proteins from the Mtb H37Rv (L4 strain) to develop the vaccine.

Antigenicity refers to the ability of the immune system ti react to the organism that the protein comes from, which is an important factor to consider when using the protein as a vaccine [87, 88]. Bacterial surface proteins are being closely studied for their potential as promising option for a vaccine candidate, as they are easily reachable and can help improve the identification of targets for treatment [89]. It is widely recognized that the primary Brucella species that causes illness in human is B. melitensis, which originally infects sheep and goats [90]. Similarly, the Euro-American Lineage 4 (33.1%; 44/133) is one of the major lineages of the Mtb prevalent in the specified area region and is commonly used in preclincial studies [8, 91]. Therefore, we selected proteins including Omp25, Omp31, MPT70, and MPT83 from these species for preliminary screening of vaccines. Moreover, it is necessary to test the homology of the target proteins. This is a critical measure to avoid the immune system mistaking the vaccine as part of the body, which could lead to the production of autoantibodies against the body’s own tissues and trigger an autoimmune reaction. Thus, after homology testing of the vaccine, the results showed that the vaccine is non-homologous to humans. This indicated that the vaccine dose not share similarities with humans. This indicates that it unlikely totrigger an autoimmune reaction or harm human tissue cells, making it a suitable target protein for additional research.

The objective of subcellular localization is to investigate the specific whereabouts of target proteins within a cell, as surface proteins are readily exposed and identifiable by the host immune system [92]. The results show that the target proteins are situated extracellularly, rendering them viable candidates for vaccine candidate proteins. Likewise, sets of target protein sequences devoid of signal peptides were chosen for the examination of cellular epitopes, as signal peptides are typically cleaved upon the nascent peptide’s entry into the endoplasmic reticulum during the translation process [93]. Therefore, the following four peptides were excluded from epitope analysis: amino acids 1–24 (MRTLKSLVIVSAALLPFSATAFAA) of Omp25, amino acids 1–20 (MKSVILASIAAMFATSAMAA) of Omp31, amino acids 1–31 (MKVKNTIAATSFAAAGLAALAVAVSPPAAA) of MPT70, and amino acids 1–25 (MINVQAKPAAAASLAAIAIAFLAGC) of MPT83.

Advancements in immunoinformatics technology have enabled the precise predicting of HLA-I/II T cell epitopes [15]. It is well understood that Human Leukocyte Antigens (HLAs) exhibit a high degree of genetic variation, with varying frequencies of different HLA alleles observed across populations of diverse ethnic backgrounds [94]. Hence, alleles exhibiting a high prevalence in regions pertinent to vaccine development were chosen for analysis. Subsequently, epitopes capable of inducing IFN-γ production were further identified to augment the capacity of Helper T lymphocytes (HTL) in eliciting an immune response within the human system. This is because IFN-γ activates the bactericidal function of macrophages, promotes antigen presentation expression and co-stimulatory molecules in the APCs, the promotion of CD8+ T cell-mediated cytotoxicity, the augmentation of apoptosis in infected macrophages, and the elimination of infected macrophages [95]. T cell epitopes should be highly associated with HLA-I and HLA-II alleles. By molecular docking, it had been demonstrated that T-cel epitopes have a high binding affinity to HLA alleles (HLA-A*02:01 and HLA-DRB1*01:01). B-cell epitopes trigger humoral immunity, producing antibodies against pathogens [96]. When antibodies attach to B-cell epitopes, they can trigger the activation of the body’s defense mechanisms, leading to various effects such as impeding the ability of bacteria to adhere to mucosal surfaces, decreasing the number of invading pathogens, and initiating complement activation [97]. Finally, by screening, we obtained 36 dominant epitopes, including 14 HTL epitopes, 13 CTL epitopes, 5 LB epitopes, and 4 CB epitopes.

The selection of suitable linkers significantly influences the proper folding, biological functionality, structural stability, and immunogenic properties of proteins [24]. AAY, GPGPG, and KK linkers are commonly constructed using flexible and hydrophilic amino acids, which have the potential to preserve the protein’s biological functionality [98]. The EAAAK linker is categorized as a rigid linker owing to its propensity to form helical structures, thereby augmenting the immunogenicity of vaccine proteins [99]. In order to form the polypeptide, it is essential to link CTL, HTL, and B-cell epitopes using Linker-AAY, Linker-GPGPG, and Linker-KK, respectively. Adjuvants play a crucial role in enhancing the antigenicity of anticipated epitopes and facilitating epitope recognition. As a result, we opted to utilize adjuvants derived from human beta-defensin 3 (hBD3) in the formulation of mRNA vaccines [99–102]. Following an immunoinformatics examination, it was determined that mRNA vaccines consist of 790 amino acids and have an atomic weight of 81 kDa. Maintaining an appropriate atomic weight is crucial in the development of vaccines that meet the specified requirement of being less than 110 kDa [103]. The calculated theoretical isoelectric point of the vaccine was found to be 9.68, indicating its predominant basic properties. Furthermore, the vaccine structure possesses a GRAVY value of -0.352, indicating its hydrophilic nature and high solubility [104]. The mRNA vaccines exhibit a reduced hydrophobicity index (GRAVY) compared to the vaccine formulated in the investigation conducted by Omoniyi et al., indicating a potentially enhanced affinity for interactions with water molecules [105]. Moreover, mRNA vaccines exhibit a half-life exceeding 10 hours in *E*. *coli*, leading to an extended duration of the vaccine’s presence immune system and thereby eliciting heightened immune responses [106]. The aliphatic index value of 73.86, surpassing the established threshold of 50.00, indicated that the vaccine demonstrates stability when exposed ti high temperatures [107]. The mRNA vaccines exhibited an instability index of 26.72, indicating a level below the threshold of 40, suggesting that the protein was stable [108]. Moreover, it is essential that vaccine proteins intended for *E*. *coli* expression exhibit solubility. The mRNA vaccines demonstrate a solubility rate of 0.93, suggesting that the vaccine structures are readily soluble within the host system. This characteristic enhances the likelihood of effective interaction with immune molecules, thereby promoting an immune response [109]. Ultimately, through the process of immunoinformatics analysis, we successfully identified a vaccine protein that exhibits high antigenicity, hydrophilicity, stability, and solubility.

The larger the proportion of random coil and extended chains within the vaccine protein composition of a vaccine facilitates the formation of antigenic epitopes [110]. The secondary structure prediction of mRNA vaccines showed 43.67% random coils and 23.67% extended chains, suggesting the formation of antigenic epitopes. The assessment of the tertiary structure model’s quality was conducted through the utilization of Z-scores and Ramachandran plots. The results indicated that the tertiary structure of mRNA vaccines conforms to the score distribution observed in the structural database for natural proteins. These findings imply that the model exhibits high quality and is suitable for subsequent analytical investigations.

Studying the molecular mechanisms involved is crucial for vaccines to effectively stimulate both cellular and humoral immunity, resulting in optimal immune responses. Toll-like receptors (TLRs) play a key role in recognizing pathogen-associated molecular patterns (PAMPs) and triggering the production of pro-inflammatory mediators, which contribute to pathogen clearance [111]. Studies have shown that TLR4 plays a crucial role in the immune response to bacterial infections [112]. The process of molecular docking was conducted for mRNA vaccines targeting TLR4 in order to simulate the immune response elicited by the vaccine within the human body. Thus, the more negative the HDOCK score, the better the docking of the complex. The docking model with the optimal docking parameters was selected for further steps.

Molecular dynamics (MD) simulation is a useful tool for studying the dynamic characteristics of mRNA vaccines and the conformational changes that occur upon binding with Toll-like receptor 4 (TLR4) by predicting the stability and intermolecular interactions of docked complexes as they evolve over a period of time. In this study, the stability of protein-ligand complexes was assessed by monitoring various parameters, including Root Mean Square Deviation (RMSD), Root Mean Square Fluctuation (RMSF), Radius of Gyration (Rog), hydrogen bonds, and Principal Component Analysis (PCA) throughout the 100 nanosecond simulation trajectories. The Molecular Mechanics Generalized Born Surface Area (MMGBSA) analysis revealed that the binding stability of the binding complex is correlated with a minimal energy requirement. Additionally, analyses of deformability, B-factor values, eigenvalue, variance plot, covariance, and cross-correlation matrices revealed negligible fluctuations. Consequently, these results provide robust evidence for the effective binding capacity of mRNA vaccines to TLR4.

The process of immune stimulation entails evaluating the ability of a vaccine to trigger the immune response in the host. In the context of mRNA vaccines, immune profiles indicate activation of various immune cells such as B lymphocytes, CTL, HTL, and macrophages responses that lead to the generation of cytokines (IFN-γ, TGF-β, IL-2, IL-10, and IL-12) as well as antibody titration (IgM, IgG1, IgG2, IgG1+IgG2, and IgM+IgG). Moreover, increased levels of natural killer cells, dendritic cells, and epithelial cells play a crucial role in the immune system’s defense mechanism. Conversely, a decrease in Treg cells may indicate a shift towards effector T cells, which could enhance the immune response [113]. Collectively, these findings indicate that mRNA vaccines have the potential to induce a robust immune reaction against B. melitensis and Mtb H37Rv (L4 strain), making them promising candidates for vaccines against brucellosis and tuberculosis.

In designing mRNA vaccines candidates, effective cloning and expression in appropriate vectors are critical stages [114]. Codon optimization plays a pivotal role in this process, given the redundancy inherent in the genetic code, allowing for the representation of numerous amino acids through multiple codons [115]. The Codon Adaptation Index (CAI) is a metric that defines a specific group of codons deemed to be most favorable and assesses their frequency within the coding regions of genes. A CAI value of 1.0 is deemed optimal, with scores exceeding 0.8 considered favorable [116]. The ideal GC content range for selecting *E*. *coli* hosts for protein expression is typically recommended to fall between 30% and 70% [117]. The present investigation demonstrated that the recombinant protein, possessing a Codon Adaptation Index (CAI) value of 1 and a GC content of 53.21, can be efficiently expressed in bacterial hosts. These findings suggest that the vaccine protein can be cloned effectively within the host organism, laying the groundwork for future research efforts.

## 5. Conclusion

*Brucella* spp. and *Mtb* spp. can cause zoonotic disease worldwide. In this study, we identified dominant T and B cell epitopes in Omp25, Omp31, MPT70, and MPT83 of *B*. *melitensis* and *Mtb* H37Rv (L4 strain) through a series of computational analyses and eventually embroidered them into multi-epitope mRNA vaccine. The recently developed vaccine exhibits favorable immunodominant characteristics and solubility. Importantly, using molecular docking, MD, and silico cloning, it was found that the mRNA vaccine -TLR4 can be stably transported in vivo and can be expressed in *E*. *coli*. Based on our findings, we believe that the vaccine candidate could be a starting point for the development of effective vaccines against both pathogens. Furthermore, it is necessary to conduct future experimental studies to confirm the immunogenicity of the mRNA vaccine.

## Supporting information

S1 Data The minimal anonymized data. (RAR)
